# Simultaneous integrated boost (SIB) to dominant intra-prostatic lesions during extreme hypofractionation for prostate cancer: the impact of rectal spacers

**DOI:** 10.1186/s13014-022-02003-8

**Published:** 2022-02-22

**Authors:** Sarah O. S. Osman, Ciaran Fairmichael, Glenn Whitten, Gavin S. Lundy, Rachel Wesselman, Melissa LaBonte Wilson, Alan R. Hounsell, Kevin M. Prise, Denise Irvine, Conor K. McGarry, Suneil Jain

**Affiliations:** 1 Patrick G Johnston Centre for Cancer Research, Queen’s University Belfast, Belfast, BT7 1NN Northern Ireland UK; 2 Radiotherapy Physics, Northern Ireland Cancer Centre, Belfast Health and Social Care Trust, Belfast, UK; 3 Clinical Oncology, Northern Ireland Cancer Centre, Belfast Health and Social Care Trust, Belfast, UK

**Keywords:** Prostate cancer, Rectal spacers, Dominant intra-prostatic lesions (DIL), Dose limiting organs

## Abstract

**Purpose:**

Boosting dominant intra-prostatic lesions (DILs) has the potential to increase the therapeutic ratio in prostate cancer radiotherapy. In this study, employing 5-fraction stereotactic ablative radiotherapy (SABR) volumetric modulated arc therapy (VMAT) to deliver 40 Gy to the prostate clinical target volume (CTV) while boosting the DIL up to 50 Gy was evaluated for patients before and after rectal spacer insertion.

**Materials and methods:**

24 Computed Tomography (CT) scans of 12 prostate cancer patients with unfavourable intermediate or high risk prostate cancer were employed in this study. At least two treatment plans were generated for each patient to compare pre- and post-spacer insertion plans. Plans were evaluated for target coverage, organs-at-risk doses, and the achievable boost dose level.

**Results:**

The CTV coverage was significantly better in plans with a spacer, V_40Gy_ 98.4% versus 97.0% (p = 0.012). Using spacers significantly reduced rectal dose in all 12 patients in this study. It was possible to boost DIL to 50 Gy to without violating dose constraints in 6 of 12 patients and to 47.5 Gy in 3 patients post-spacer insertion. For 3 patients (25%) it was not possible to boost DIL above 45 Gy even with a spacer in situ. Without a spacer, for 6 patient (50%) clinically acceptable plan were only achieved when the DIL dose was lowered to 45 Gy. In five of these 6 patients the dose limiting structure was the urethra (urethra planning risk volume V_45Gy_ [cc] ≤ 0.1 cc constraint).

**Conclusions:**

Clinically acceptable plans for 5 fraction SABR, 40 Gy to the prostate CTV, with a SIB to DIL (45–50 Gy) were achieved. The boost dose achieved was DIL location dependent and primarily affected by DIL’s proximity to the urethra. Compared to plans before spacer insertion, higher DIL dose were achieved with spacer in situ for 25% of the patients. Moreover, significant reduction in rectal dose and better target coverage were also achieved for all patients with spacers in situ.

**Supplementary Information:**

The online version contains supplementary material available at 10.1186/s13014-022-02003-8.

## Introduction

Hypo-fractionation is an attractive option in the management of prostate cancer not only because it is cost-effective and convenient for patients, but also due to the potential therapeutic benefits. There is mounting evidence suggesting that prostate cancers are highly sensitive to dose per fraction rather than the total dose [1–3]. Clinical trials of moderate and extreme hypo-fractionation have demonstrated the feasibility and non-inferiority of hypo- vs conventional-fractionation [4–6].

Local recurrences post-radiotherapy mostly originate from highly radio-resistant sub-volumes within the primary treated volume (dominant intra-prostatic lesions (DILs)) [7–9]. Boosting DILs to significantly higher doses has the potential to maximize tumour control [10]; however, this is constrained by the increased risk of toxicity. The proximity of the prostate to the rectum and the bladder makes DIL boosting more challenging, as both are radiosensitive organs that are susceptible to movement and/or deformations.

Phase 3 trials examining integrated boosts up to 95 Gy in standard fractionation (77 Gy in 35 fractions to the entire prostate) have shown acceptable toxicity [11] and further phase 3 trials are currently underway in moderately hypo-fractionated treatments [12]. A number of phase 1 and 2 trials of integrated boosts during stereotactic ablative radiotherapy (SABR) have demonstrated acceptable toxicity [13–17].

In recent years, anatomy modifiers of different types have been introduced to spare the rectum by separating the anterior rectal wall from the prostate. Most widely used techniques include injected hydrogel spacers, hyaluronic acid or saline-filled balloons [18–21].

As demonstrated in many studies, rectal spacers are well tolerated, cost effective and they reduce rectal dose, leading to reduced RT related acute and late toxicity [18, 21, 22]. However, there is little information available on the combination of rectal spacer devices with SABR DIL boosts.

In a previous study [21], we presented our first clinical experience of treating high-risk prostate cancer patients with SABR-VMAT in combination with rectal spacers. Insertion of a spacer resulted in a clinically and statistically significant reduction in rectal doses for all patients investigated [21].

The aim of this study was to assess the technical feasibility of delivering 5-fraction SABR with a SIB for high-risk prostate cancer patients. The impact of using rectal spacers was evaluated by comparing plans generated using planning CT scans of patients taken before and after spacer insertion. Our hypothesis is that when rectal spacers are used, it is possible to boost all intra-prostatic lesions to 50 Gy without increasing the risk of toxicity. A comparison was made between pre-spacer and post-spacer insertion plans in terms of dose coverage and the dose received by organs at risk (OARs). The effect of the location of the DIL region on the achievable boost dose level was also evaluated.

## Materials and methods

### Ethical approval and patient selection

All patients included in this study were treated in the SPORT High-Risk clinical trial evaluating SABR to prostate and/or pelvic lymph nodes (ClinicalTrials.gov Identifier: NCT03253978). The SPORT trial was approved by the Health and Social Care Research Ethics Committee (REC) (REC reference 15/NI/0192).

This study included patients with histologically confirmed prostate adenocarcinoma presenting with unfavourable intermediate risk (Gleason 4 + 3) or high risk localised prostate cancer clinical stage (T3a N0 M0, Gleason score 8–10 and/or PSA > 20) and who were planned to receive 1–3 years ADT as part of their standard treatment. Patients with overt T3b stage cancer were excluded from the trial (Full details NCT03253978). 24 CT scans from 12 consecutive patients with visible DILs on their diagnostic MRI were retrospectively used for this study. Any patients who did not have a visible index lesion were excluded.

### Hydrogel spacer, fiducial marker implantation, image acquisition and segmentation

Each patients had a spacer (SpaceOAR™, Augmenix, Waltham, MA) and 3 fiducial markers implanted under local anaesthetic. All patients had pre- and post-spacer CT scans acquired using a helical CT-simulator (2.5 mm slice thickness, 0.9766 × 0.9766 mm^2^ pixel size). Patients were instructed to follow bowel and bladder preparation protocols before CT acquisition and each treatment session [21].

The post-spacer CT and MR scans were acquired 1 week after spacer and fiducial marker insertion. MR scans were fused with the CT scans using the Eclipse™ treatment planning system v. 15.7 (Varian Medical Systems, Palo Alto, CA) for contouring. The pre- biopsy diagnostic multiparametric MR images assisted the delineation of dominant intra-**p**rostatic **b**oost lesions (GTVpb), while the post-spacer MR images were used for other structures, including urethra and spacers on post-spacer scans. Urethra was contoured using T2 weighted axial, sagittal and coronal images. Structures of interest were contoured manually in Eclipse by one of two consultant clinical oncologists, where the treating oncologist contoured the same structures on both CT image sets to allow comparison between pre- and post-spacer insertion plans. In this study, only the prostate and proximal 10 mm of the seminal vesicles (PSV) CTV was used (referred to as Tpsv hereafter), reflecting the “prostate only” trial arm. A suitable DIL was defined as a lesion with a score 4 or 5 on the PI-RADS v2 scheme [23] measuring greater than 5 mm.

Other OARs contoured were the rectum, bladder, femoral heads, bowel, sigmoid and penile bulb.

### Treatment planning

Three target volumes with three different dose levels were defined: (1) Tpsv which was prescribed 40 Gy; (2) PTVpsv, which was generated by expanding the Tpsv by 5 mm isotropic margins, was prescribed 36.25 Gy; (3) the CTVpb, generated by expanding the GTVpb with 3 mm margins isotropically (CTVpb was cropped if extended beyond the Tpsv contour), which was initially prescribed a dose of 50 Gy.

All dose levels to be delivered simultaneously in 5 treatment fractions on alternate days (over no more than 14 days). A planning organ at risk (PRV) urethra structure was constructed by expanding the prostatic urethra with a 3 mm isotropic margin and the CTVpb was cropped again to remove any overlap with the urethra_PRV.

All planning was performed in Eclipse using the VMAT Photon Optimizer (v. 15.6) and Acuros XB dose calculation (v. 13.6.23) algorithms for a Varian TrueBeam Linac. The dose calculation grid size used was 2.5 mm, the heterogeneity correction was switched on and dose-to-medium reporting was enabled.

The goal of the treatment plans was to deliver DIL dose up to 50 Gy whilst meeting the specified normal tissue constraints (Table 1). When the mandatory OARs constraints were not met, the prescribed dose to the DIL was incrementally dropped by 2.5 Gy increments to 47.5 Gy and 45 Gy as the lowest boost dose level. Two full VMAT arcs of 10 MV flattening-filter-free photon beam with a maximum dose rate of 2400 MU/min were used [24].

**Table 1:** Dose-volume objectives and constraints for the SPORT trial adopted in this study, minor and major variations, definitions and the achieved median (range) dose levels and plan quality metrics, and NTCPs

Target volumes	Objectives/constraints	Minor variation	Major variation	Pre-spacer	Post-spacer
CTVpb	V_Px_ [%] ≥ 95%	95% > V_Px_ ≥ 90%		95.72 (90.86–99.97)	97.88 (92.44–99.98)*
	medianD [Gy]			47.77 (46.16–52.50)	47.77 (46.14–52.40)
	D_2%_ ≤ 55 Gy			48.93 (46.20–52.60)	48.55 (47.00–52.60)
Tpsv	V_40Gy_ [%] ≥ 95%	V_40Gy_ ≥ 90%	V_40Gy_ < 90%	96.98 (93.12–99.31)	98.40 (95.89–99.81)*
PTVpsv	V_36.25 Gy_ [%] ≥ 95%	V_36.25 Gy_ ≥ 90%	V_36.25 Gy_ < 90%	95.41 (91.28–97.02)	95.93 (94.60–99.79)
	D_98%_ [Gy] ≥ 34.4 Gy			35.37 (34.50–35.90)	35.60 (35.00–37.20)*
	D_max_ [%] < 120% of boost			106.12 (104.67–109.34)	106.28 (105.04–111.38)
	D_2%_ [%] ≤ 107% of boost			103.19 (101.00–104.67)	103.70 (102.20–106.02)

Organs at risk	Objectives/constraints	Minor variation	Major variation	Pre-spacer	Post-spacer
Rectum	V_18.1 Gy_ [%] < 50%			29.42 (18.16–42.35)	23.22 (3.43–34.99)**
	V_29Gy_ [%] < 20%			12.59 (5.69–19.40)	5.48 (0.01–13.30)**
	V_36Gy_ [cc] < 1 cc	< 2 cc	> 2 cc	1.60 (0.51–2.00)	0.42 (0.00–1.00)**
EUD [Gy]	EUD [Gy]			59.42 (55.37–65.11)	52.24 (24.30–57.40)**
NTCP [%]	NTCP [%]			4.05 (1.75–11.46)	0.86 (0.00–2.72)**
Bladder	V_18.1 Gy_ [%] < 40%			12.67 (5.35–22.33)	11.29 (7.37–21.83)
	V_37Gy_ [cc] < 10 cc	10 cc ≤ V_37Gy_ < 20 cc	V_37Gy_ ≥ 20 cc	6.42 (0.72–9.33)	5.22 (0.43–8.39)
Urethra	V_42Gy_ [%] < 50%			19.12 (0.03–49.81)	19.59 (0.00–47.06)
	V_45Gy_ [cc] < 0.01 cc			0.00 (0.00–0.00)	0.00 (0.00–0.00)
Urethra_PRV	V_42Gy_ [%] < 50%			27.82 (9.76–47.16)	25.30 (9.52–44.04)
	V_45Gy_ [cc] < 0.1 cc			0.04 (0.00–0.10)	0.01 (0.00–0.08)
Plan	CI			1.15 (1.04–1.22)	1.18 (1.11–1.31)
	R50%			3.53 (3.15–3.87)	3.58 (3.37–4.11)

V_xGy_ = volume receiving × Gy, EUD = equivalent uniform dose, RTOG conformity index $\left( {{\text{CI}} = \frac{{{\text{Volume}}\;{\text{ of}}\;{\text{ 95}}\%\;{\text{isodose}}}}{{{\text{PTV }}\;{\text{volume }}}}} \right)$, ${\text{R}}_{50} = \frac{{{\text{Vol}}_{{50\% \;{\text{pres}}}} }}{{{\text{PTV}}\;{\text{ volume}}}}$, where ${\text{Vol}}_{{{\text{50}}\%\;{\text{pres}}}}$ is the tissue volumes receiving at least 50% of the PTV prescription dose. All patients’ pre- and post-spacer plans were generated using matched boost dose levels* indicates a p-value ≤ 0.05 and ** indicates a p-value ≤ 0.01. Bladder and rectum volume in pre- and post- spacer scans are provided in Additional file 1: Table ST3.

### Plan dosimetric and biological evaluation

The goal of plan evaluation was firstly to identify on which scans it is possible to plan SIB of 50 Gy to the DIL without violating OARs constraints. Secondly, an analysis of achieved dose levels pre- vs post-spacer insertion was conducted. Finally, to assess the effect of using rectal spacers, all patients were planned using matched dose levels on pre- and post-spacer scans (e.g. if the DIL dose achieved on a pre-spacer plan was 45 Gy and > 45 Gy post-spacer, plans with 45 Gy DIL dose were generated for both pe- and post spacer). The dose metrics described in Table 1, dose conformity index [25], and the medium-dose spillage outside the PTV were used to evaluate the plans generated on the two CT image sets. Included in Table 1 are definitions for minor and major variations from the dose objectives considered in this investigation. In all the plans, the goal of the optimization was to maximize the coverage of the targets without introducing any major violations in OARs constraints.

Comparative statistical analysis of pre- and post-spacer insertion plans was conducted in R version 3.6.3 using the non-parametric two-sided paired-sample Wilcoxon signed rank test at a p ≤ 0.05 significance level.

Additionally, tumour control probabilities (TCPs) were calculated using the linear quadratic (LQ)-Marsden model [26] implemented in RADBIOMOD version 0.3 [27], and NTCPs (Grade ≥ 2 rectal bleeding) were calculated using the Lyman–Kutcher–Burman (LKB) model [28], Additional file 1: S1.

### DIL location and the achieved DIL dose level

The location of the GTVpb volumes and the achieved dose levels were described with guidance of the PI-RADS™ scheme v2 [23]. Moreover, to assess the proximity of the DIL to the urethra_PRV, an assessment of the overlap volume of the original uncropped CTVpb (GTVpb + 3 mm isotropic margins) and urethra_PRV was made. Additionally, the treated CTVpb was extended by 1 mm isotropically and the overlap with the urethra_PRV, rectum and bladder was assessed to give an indication of the complexity of the treatment plan (i.e. proximity of OAR to the CTVpb).

## Results

The median DIL CTVpb volume was 4.4 cm^3^ (range 1.9–7.6 cm^3^) and the median PTVpsv volume was 111.1cm^3^ (range: 73.9–133.0 cm^3^).

**Fig. 1:**
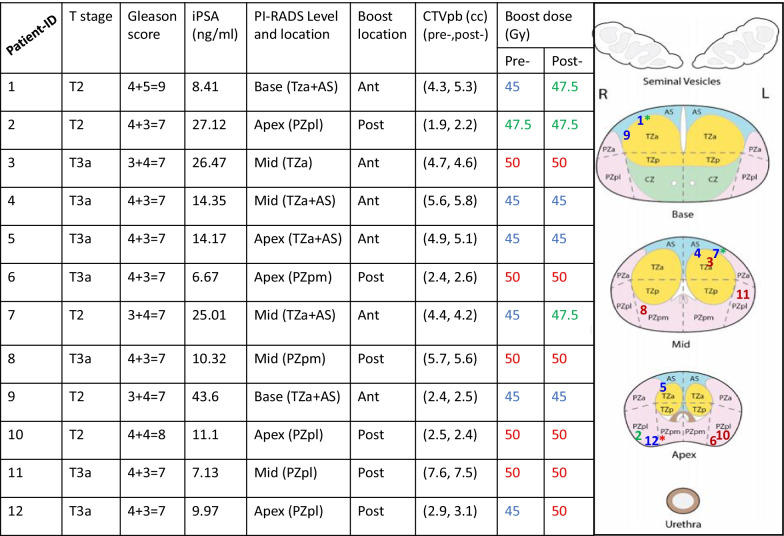
An overview of the twelve patients’ boost volume location (ant = anterior, post = posterior), location on PI-RADS™ v2 scheme and the associated achieved dose levels on pre-spacer (pre-) and post-spacer (post-) plans. * The asterisks on the numbers on the PI-RADS figure indicate that a higher dose level was achieved in post-spacer plans

Figure 1 gives an overview of staging information, the location of the DIL volumes within the prostate, and the achieved SIB dose levels for all 12 patients in this study.

In 25% of the patients (3/12), higher DIL boost doses were possible in post-spacer compared to pre-spacer plans. The median achieved dose pre-spacer was 45 Gy and 50 Gy in post-spacer insertion plans.

Using pre-spacer scans, clinically acceptable plans were achieved with a SIB of 50 Gy for 5 patients, 47.5 Gy for one patient and 45 Gy for 5 patients. For patient 1, even when planning with a 45 Gy SIB it was not possible to meet rectal dose constrains (V_29Gy_ = 21.8%, V_36Gy_ = 2.6 cc). This was due to the 36.25 Gy dose coverage requirement to the PTV rather than the boost dose, which was not an issue for the post-spacer plans.

For the post-spacer scans, it was possible to generate clinically acceptable plans with 50 Gy SIB to the DILs for 6 patients, 47.5 Gy for 3 patients and SIB of 45 Gy in 3 patients. Therefore, the achievable SIB dose increased from 45 to 47.5 Gy in 2 patients and from 45 to 50 Gy in 1 patient (represented by * in Fig. 1).

In all anterior DIL plans, both with and without spacers, the dose-limiting organ was the urethra_PRV. For posterior DIL plans, without a spacer, the dose limiting-organ was the rectum, while with spacers-in-situ, all rectal the dose-volume constraints were always met (i.e. no minor or major violations observed). In this study, the DIL dose affected the rectum in only one patient (patient 12 with a posterior DIL) and using a spacer allowed boosting of the dose to 50 Gy compared to 45 Gy pre-spacer.

**Fig. 2:**
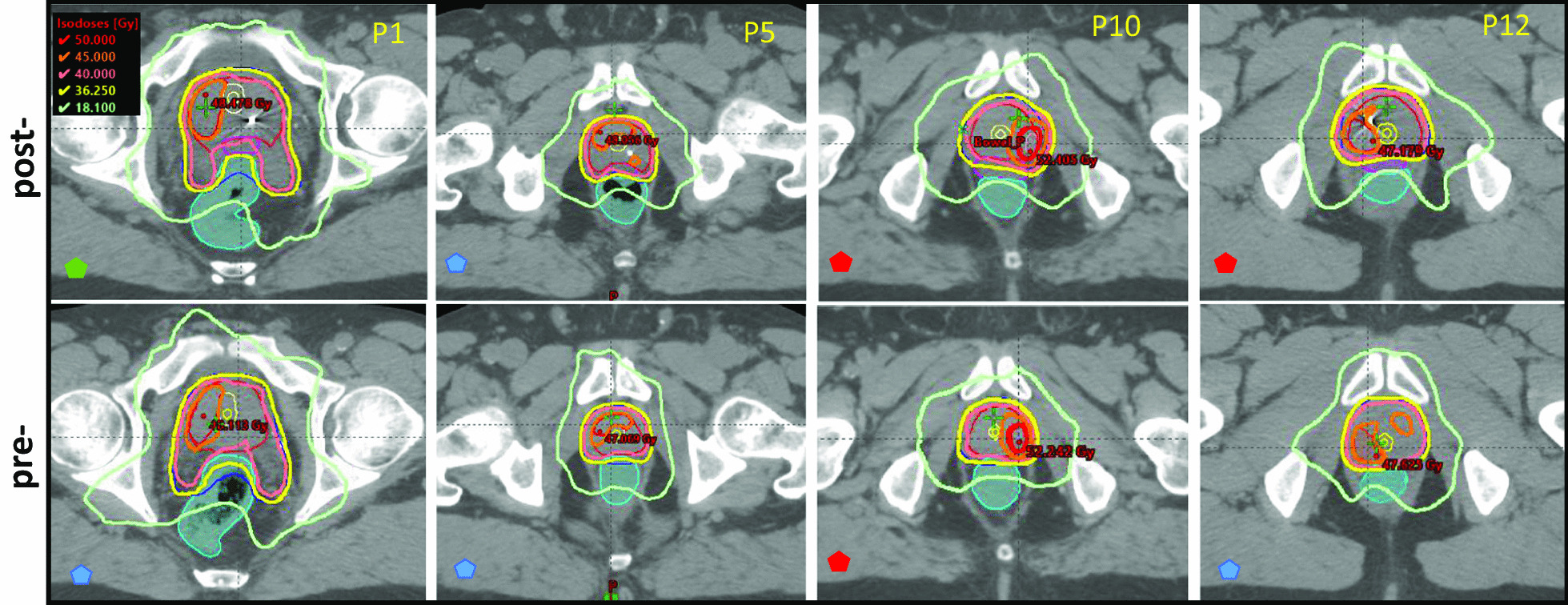
Example axial views of pre- and post-spacer CT scans and isodose levels for matched dose plans. The prescribed dose to the prostate Tpsv (shown in red) was 40 Gy, the PTVpsv (blue) was 36.25 Gy and the CTVpb (outlined in red). The coloured geometrical shapes (pentagons) on the lower left side of the figures indicate achievable DIL dose level; 50 Gy (red), 47.5 Gy (green) or 45 Gy (blue). Urethra and urethra_PRV are outlined yellow, rectum is cyan and the hydrogel spacer in is outlined in magenta colour. The other lines indicate selected iso-dose lines as indicated by the key

To assess the effect of spacer on each plan, all patients were finally planned using matching dose levels (Table 1). For example, for patient 1 the boost dose was lowered to 45 Gy in the post-spacer plan to facilitate the comparison with the pre-spacer plan (Fig. 2). Figure 2 shows example transverse slices at the level of the DIL volume (CTVpb) for representative patients (pre- and post-spacer insertion). A similar figure for all the patients in the study is presented in Additional file 1: Figure SF1. All isodose lines shown in Fig. 2 were generated for matched CTVpb dose prescription (pre- and post-spacer). The achieved boost dose level is indicated by the coloured geometrical shapes shown at the bottom left of each scan as described in the figure legend.

With a spacer in-situ, the rectal V_36Gy_ was always below the optimal constraint of 1 cc (Table 1). As expected, rectal dose constraints were harder to meet for the same patients in pre-spacer plans. This was particularly the case for patients with noticeable overlap of the PTVpsv (prescribed to 36.25 Gy) and the rectum (Fig. 2, SF1). The priority of the treatment plan was to achieve target coverage while meeting all OARs dose constraints (Table 1). Therefore, in pre-spacer plans, only minor variations in OAR constraints were allowed, resulting in more reduction in targets coverage as allowed by planning protocols.

**Fig. 3:**
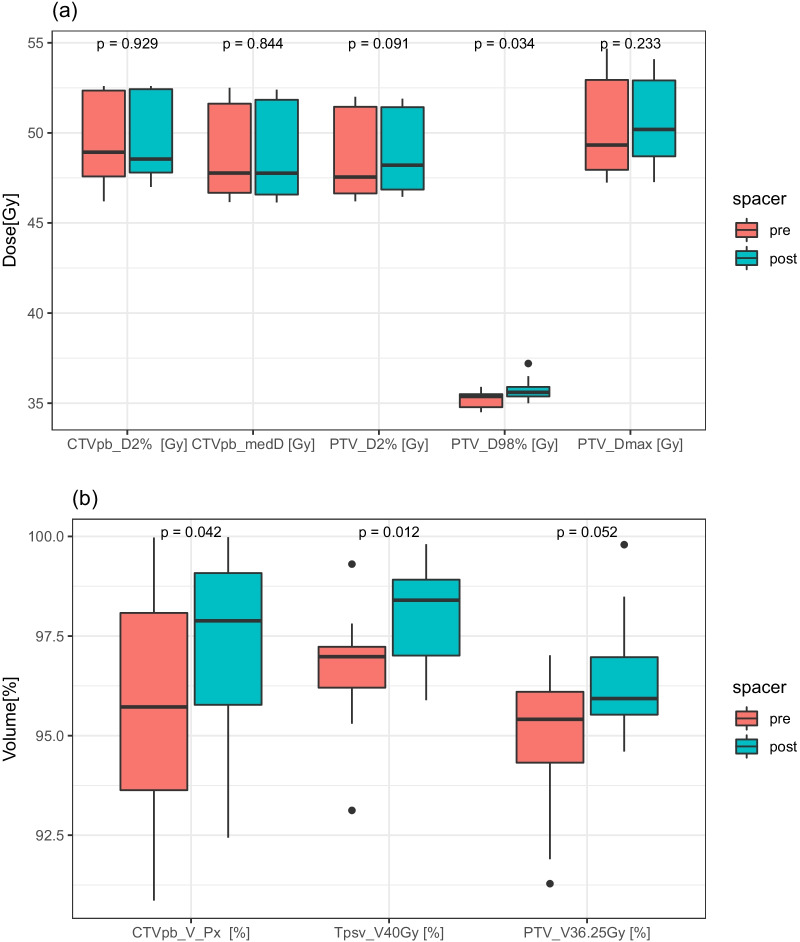
Box plots of targets’ **a** absolute doses [Gy] achieved for pre- and post-spacer plans, **b** percentage volumes of the CTVpb, Tpsv, and PTVpsv receiving specified prescription doses. The median values are indicated by the central line within the box, and the edges of the box represent the inter-quartiles range, the minimum, maximum values (excluding outliers; any values over 1.5 times the interquartile range over the 75th percentile or any values under 1.5 times the interquartile range under the 25th percentile) are represented by the whiskers, and the outliers are plotted as individual points. p values < 0.05 are considered significant

**Fig. 4:**
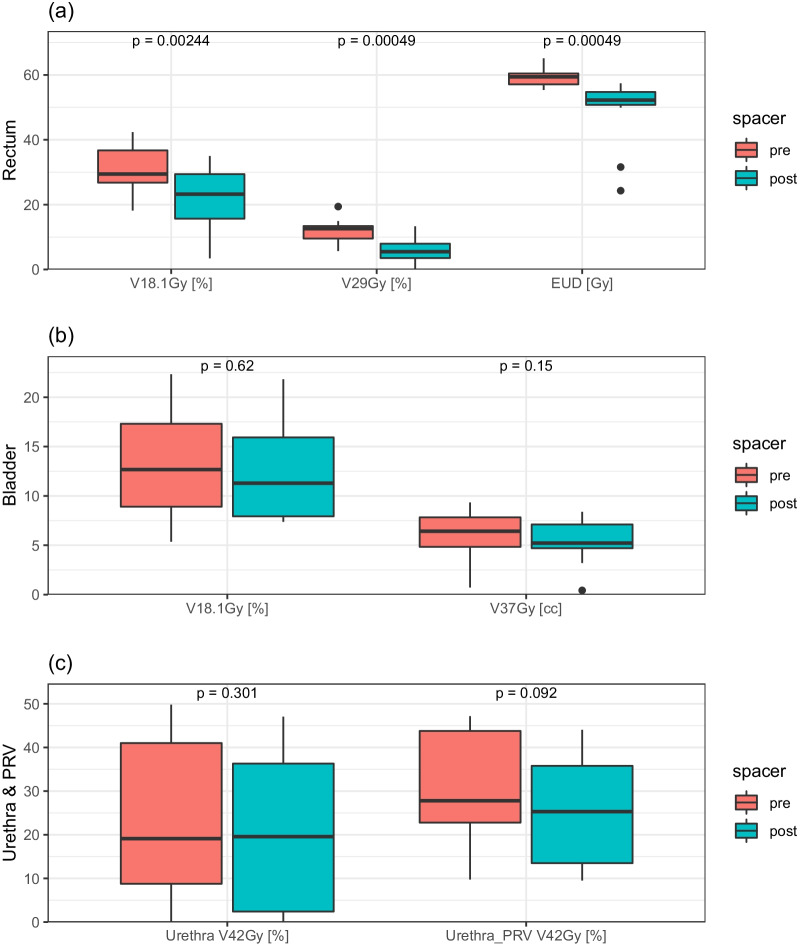
Box plots showing OARs **a** rectum, **b** bladder and **c** urethra and urethra_PRV doses achieved for pre- and post-spacer plans. The median values are indicated by the central line within the box, and the edges of the box represent the inter-quartiles range, the minimum, maximum values (excluding outliers) are represented by the whiskers, and the outliers are plotted as individual points. p values < 0.05 are considered significant

Table 1, Figs. 3 and 4 show comparisons of target coverage and OARs dose when patients were planned with and without rectal spacers. The CTVpb and Tpsv coverage were slightly, but significantly better in post-spacer plans compared to pre-spacer plans. No significant differences were observed in TCP between pre- and post- spacer plans, TCP_Boost_ = 98.3% versus 98.0%, TCP_Tpsv-Boost_ = 97.2% versus 97.3%, respectively, Additional file 1: S1 and Additional file 1: Figure SF2.

All rectal dose metrics and the associated NTCP (grade 2 + rectal bleeding) values were significantly lower in post-spacer plans compared to plans generated on pre-spacer scans (NTCP = 0.9% [0–2.7%] vs 4% [1.8–11.5%] (p = 0.00049), respectively). The dose received by the bowel, sigmoid, femoral heads, and penile bulb were minimal in all plans pre- and post-spacer insertion.

In post-spacer plans, the dose limiting structures were the urethras (V_45Gy_ < 0.01 cc) and the urethra_PRVs (V_45Gy_ < 0.1 cc). A quantitative analysis of CTVpb overlap with urethra_PRV and the corresponding achieved DIL dose level are presented in Additional file 1: Table ST1.

Two observations from the data presented in Additional file 1: Table ST1 warranted further investigation. Firstly, for patient 7 with frontal DIL (Additional file 1: Figure SF1), there was more overlap between CTVpb and urethra in post-spacer scans. Reviewing the CT scans, (Additional file 1: Figure SF3) it appears that the spacer is causing an anterior translation of the urethra bringing it closer to the CTVpb. Secondly, for patient 12 with the posterior-lateral DIL, there was a relatively large overlap in pre-spacer plan but not post-spacer. Inserting the spacer appears to have pushed the urethra away from the CTVpb. Of particular note for this patient, a SIB dose of 50 Gy was achieved post-spacer but only when lowering the dose to 45 Gy in pre-spacer plans were all dose constraints met. To quantify these effects, we measured the dimensions of the prostate at the centre of the PTVpsv on pre- and post-spacer scans for these patients (Additional file 1: Table ST2). The insertion of the spacer reduced the anterior–posterior dimension from 3.1 to 2.6 cm in patient 7 and 3.8 to 3.3 cm in-patient 12. An increase of the lateral dimensions of the prostate from 4.2 to 4.4 cm (patient 7) and 3.8 to 4.0 cm (patient 12) was observed.

## Discussion and conclusions

In this planning study, we assessed the feasibility of delivering high dose SIB to DILs in the context of prostate SABR before and after rectal spacer insertion. The aim was to deliver the boost dose while keeping OARs’ doses below the specified SABR dose limits in order to maximize the therapeutic ratio of the treatment without adversely affecting patients’ quality of life.

For all the patients in this study, spacer insertion significantly reduced rectum dose metrics and NTCP values compared with plans generated without rectal spacers as shown in Table 1, Fig. 1, and Additional file 1: Figures SF1 and SF2. Using a rectal spacer also enabled an increase in DIL boost dose in 25% of the patients. In pre-spacer plans, the coverage of the PTV and the Tpsv were also compromised in some cases to meet rectal dose constraints. However, this did not translate to significant differences in the TCP values for either the boost or the Tpsv excluding boost CTV in post- and pre-spacer plans.

In 50% of the patients in this study, the dose limiting organs were the urethra and the urethra_PRV. This was highly influenced by the location of the DIL and its proximity to the urethra [29]. The median boost dose was 45 Gy for anterior tumours, and 50 Gy in posterior tumours. An analysis of the uncropped CTVpb (GTVpb + 3 mm) overlap with urethra_PRV demonstrated that this measure could be indicative of the achievable boost dose level (Additional file 1: Table ST1). It can be seen from this table that an overlap of ≥ 0.1 cc was indicative of the need for a lower boost dose. Even when extending the CTVpb by a 1 mm margins, there was no overlap with the rectum or the bladder in 92% of the patients.

This study shows that the achieved boost dose level is strongly influenced by the location of the boost volume, with anterior DIL volumes being more challenging to boost to doses greater than 45 Gy. Contrary to our hypothesis, even when spacers were used, only in 50% of the study cohort was it possible to generate clinically acceptable plans with a 50 Gy SIB to DIL. Interestingly, for patients with posteriorly located boosts, target and OARs doses were easier to achieve even without a spacer. This could be a result of limiting the boost volume to the Tpsv and consequently ensuring there is no overlap between the CTVpb and the rectum, regardless of the presence of a rectal spacer.

There does not appear to be a consensus on appropriate margins for DIL boost volumes; margins in other SABR studies have ranged from 0 to 5 mm [15, 30]. Urethral constraints and PRV margins currently employed are also variable and the challenges in boosting anterior areas would be eased with less strict urethral constraints. Other clinical trials have used higher maximum doses to the urethra [30], smaller or no urethral PRVs [13, 15] or excluded DILs within 3 mm of the urethra [31]. However, even with the tight constraints used in this study, we were able to achieve at least a planned dose of 45 Gy in all patients (except on one patient on pre-spacer scan). This remains a significant dose escalation: 45 Gy, 47.5 Gy and 50 Gy are the equivalent of 135.0 Gy, 149.3 Gy and 164.3 Gy in 2 Gy fractions respectively when converted using the linear quadratic equation and an (α/β = 1.5).

Other studies have commented on the boost dose limitations imposed by rectal constraints [13, 15]. This study did not find this to the same extent, but 6 of the 12 cases analysed had DILs positioned in the anterior prostate where the benefit of a rectal spacer is likely to be minimal. Despite this, plans following spacer insertion had improved PTVpsv coverage and lower rectal NTCP.

In this study, it was observed that spacer insertion seemed to deform the prostate (i.e. changes in the anterior–posterior and the left–right dimensions). These changes are in-line with our observations of urethral shifts. These observations warrant future investigations of the dosimetric impact of prostate deformations due to rectal spacer insertion in relation to the location of DILs. Furthermore, when outlining the GTVpb, such changes are a reminder that the diagnostic MRI should only be used as a guide to DIL size and position: the most accurate outline will adapt the MRI volume to take into account any change in the overall shape of the prostate (e.g. due to hormone therapy or spacer insertion).

Rectal spacer positioning was symmetrical across the midline for all but one patient, Additional file 1: Figure SF4 (patient 8). For this patient, the spacer was predominantly on the same side as the DIL boost volume. However, if the boost volume was on the contralateral side to a posterior DIL, planning would have been much more complicated; suggesting the accuracy of spacer placement plays a key role, particularly for boosts to posterior DILs.

The authors acknowledge that this study has some limitations. Firstly, it can be argued that generous margins were applied for the urethra with strict dose constraints. In this study, fiducial markers-based daily-online-image-guidance with CBCT (pre- each arc delivery and post-treatment) will be employed, however, the urethra is not visible on CT scans. Another option would be to use a urethral catheter with contrast medium although this is an invasive approach. Therefore, the use of PRV was necessary to account for inter- and intra-fraction movements compared to urethral position on planning CT scan. These margins could be revised with the introduction of MR-based-image-guidance and MRI-linacs [32, 33]. To realise the benefit of SABR further, other IGRT strategies that were found effective in monitoring and reducing intra-fraction motion (e.g. gating and real time tracking) may also be adopted to improve the accuracy of the dose delivered to the target and organs at risk [34, 35]. A second limitation is that, despite the variable locations of the DILs volumes investigated in this study, the small number of patients makes it difficult to generalise our conclusions. Yet, this study demonstrates the feasibility delivering 45–50 Gy SIB to DIL without increasing the risk of toxicity in some of the patients. The results of this study will be validated prospectively in future patients. Finally, while comparing radiotherapy plans before and after spacer insertion allowed each patient to be their own matched control, it was not possible to compare clinical outcomes from treatment with and without rectal spacers as all treatments were delivered with the rectal spacer in-situ.

This study shows that boosting dominant intra-prostatic lesions up to 50 Gy without increasing the risk of normal tissue complications is technically feasible for selected patients. The dose-limiting organ in these cases is the urethra. Rectal spacers are favourable for all patients with some patients benefiting more than others. This depends not only on the anatomy of the patients but also on the way rectal spacers are positioned.

## Supplementary Information

**Additional file 1**. Supplementary material.

## Data Availability

The datasets used and/or analysed during the current study are available from the corresponding author on reasonable request.

## Abbreviations

SIB: Simultaneous integrated boost
EBRT: External beam radiotherapy
IMRT: Intensity modulated radiotherapy
SABR: Stereotactic ablative body radiation therapy
VMAT: Volumetric modulated arc therapy
IGRT: Image guided radiotherapy
DIL: Dominant intra-prostatic legion
PRV: Planning organ at risk
TCP: Tumour control probability
NTCP: Normal tissue complication probability
FM: Fiducial marker
ROI: Region of interest
CBCT: Cone beam CT
OAR: Organs at risk
PSA: Prostate-specific antigen
SPORT: Stereotactic prostate RT in high-risk localised prostate cancer with or without elective nodal irradiation
