# Supplementary material for: Simultaneous integrated boost (SIB) to dominant intra-prostatic lesions during extreme hypofractionation for prostate cancer: the impact of rectal spacers
Source: Radiat Oncol. 2022 Feb 22;17:38. doi: 10.1186/s13014-022-02003-8 (PMC8862253; doi:10.1186/s13014-022-02003-8)

**Supplementary material**

**S1- TCP and NTCP parameters**

In this analysis, the $\frac{\alpha}{\beta}$ ratio was chosen to be$1.5 Gy$. The TCP was calculated for the prostate gland structure excluding the DIL volume (${TCP}_{P-boost}$) and for the DIL separately (${TCP}_{boost}$). The clonogenic cell density $\rho_{Clon}$ in the non-DIL prostate was assumed to be $6.2\times{10}^{4}{cm}^{-3}$ and for the DIL $\rho_{Clon}$ was assumed to be $1\times{10}^{7}{cm}^{-3}$. The radiosensitivity of the clonogenic cells $(\alpha)$ is assumed to vary according to a Gaussian distribution with a mean $\bar{\alpha}= 0.217 {Gy}^{-1}$ and a standard deviation $\sigma_{\alpha}= 0.082 {Gy}^{-1}$ [1]. For comparing plans, the simplest version of this model was used (not including other radiobiological factors e.g. hypoxia, radio-sensitization or repopulation).

NTCPs were calculated using the Lyman–Kutcher–Burman (LKB) model [2]. The LKB parameters used for assessing Grade≥2 late toxicity or rectal bleeding were;$n=0.09, m=0.13, {TD}_{50}=76.9 Gy$ [3].

**References:**

[1] Nahum AE, Uzan J. (Radio)biological optimization of external-beam radiotherapy. Comput Math Methods Med 2012;2012. https://doi.org/10.1155/2012/329214.

[2] Gay HA, Niemierko A. A free program for calculating EUD-based NTCP and TCP in external beam radiotherapy. Phys Medica 2007. https://doi.org/10.1016/j.ejmp.2007.07.001.

[3] Michalski JM, Gay H, Jackson A, Tucker SL, Deasy JO. Radiation Dose-Volume Effects in Radiation-Induced Rectal Injury. Int J Radiat Oncol Biol Phys 2010;76:123–9. https://doi.org/10.1016/j.ijrobp.2009.03.078.

**Supplementary table ST1: Volume overlap in [cc] of the original uncropped boost CTV (O_CTVpb = GTVpb+3mm isotropic margin) with the urethra PRV (U_PRV) in pre- and post- spacer scans. CTVpb was extended by a 1mm isotropic margin and the overlap with U_PRV, rectum and bladder is reported in volume [cc]. Achieved dose levels were 50Gy (red bold), 47.5 (green), 45Gy (blue italic).**

**Supplementary table ST2: The size of the prostate at the centre of the PTVpsv was assessed by measuring the anterior-posterior and the left-right dimensions of the prostate in pre- and post-spacer scans.**

**Supplementary table ST3: Bladder and rectal volumes in pre- and post-spacer scans.**

**Supplementary Figure SF1: Axial views of pre- and post-spacer CT scans and dose levels for matched dose plans (patients1−12). The dose prescribed to the prostate Tpsv (shown in red) was 40 Gy, the PTV (blue) was 36.25 Gy and the CTVpb (outlined in red). The coloured geometrical circle on the lower left side of the figures indicate achievable DIL dose level; 50 Gy (red ), 47.5 Gy (green) or 45 Gy (blue). Urethra and urethra_PRV are outlined yellow, rectum is cyan and the hydrogel spacer in is outlined in magenta colour. The other lines indicate selected iso-dose lines as indicated by the key.**


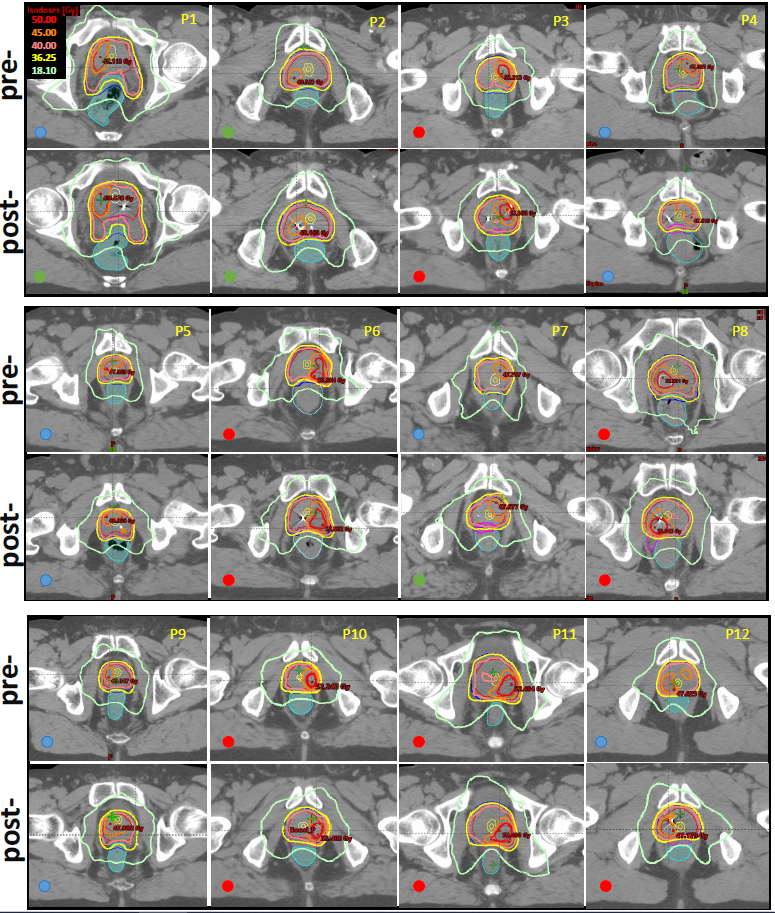


**Supplementary Figure SF2: Box plots of (a) percentage volumes of targets and the expected tumour control probability (TCP) calculated for the boost CTVpb and for the prostate excluding boost structure, (b) doses received by the rectum, the equivalent uniform dose, and the % NTCP (end-point rectal bleeding grade +2) in pre- and post-spacer plans. The median values are indicated by the central line within the box, and the edges of the box represent the inter-quartiles range, the minimum, maximum values (excluding outliers) are represented by the whiskers, and the outliers are plotted as individual points. p values <0.05 are considered significant.**

(a)


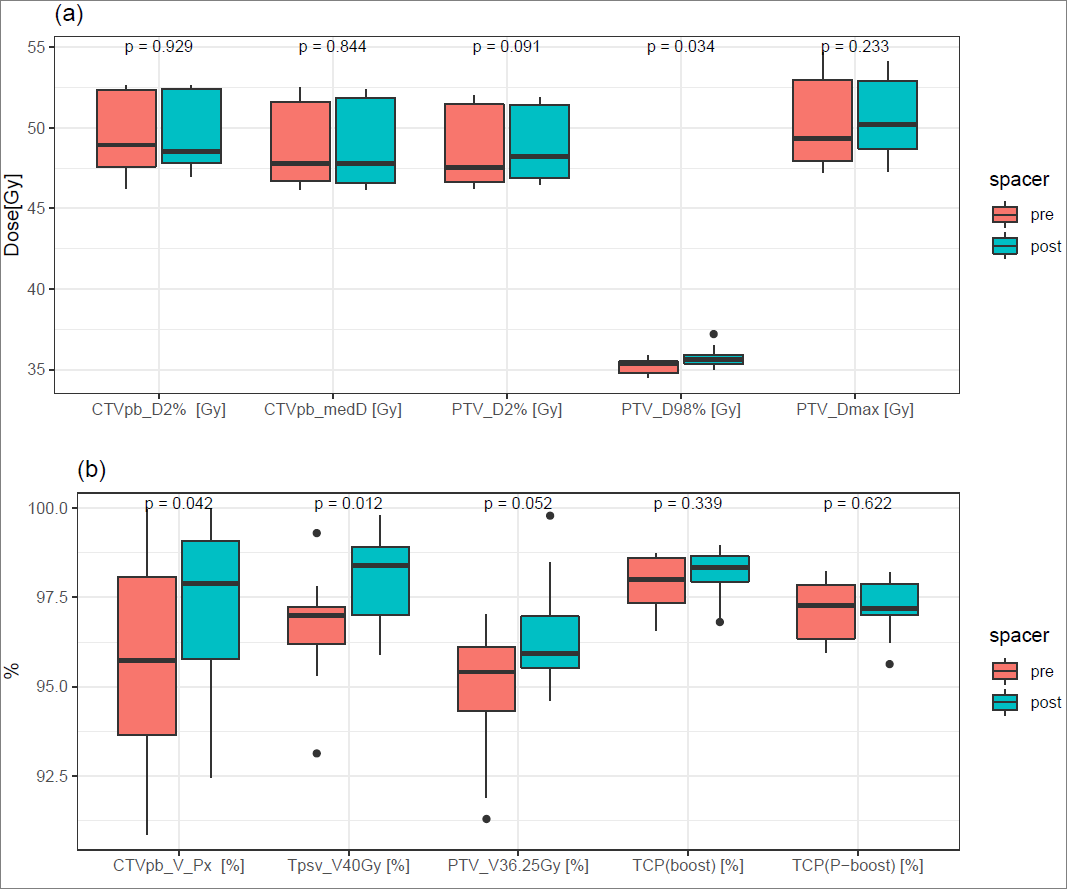


(b)


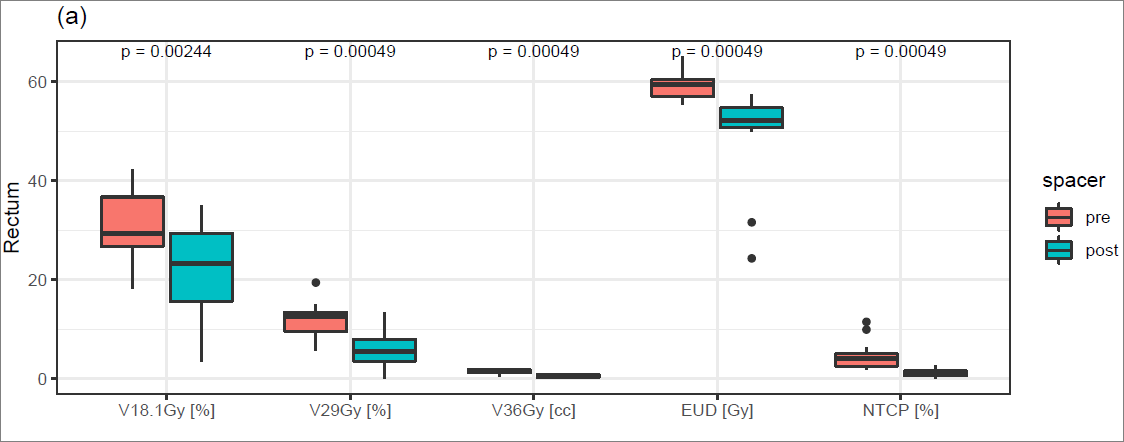


**Supplementary Figure SF3: An overlay (toggle colour blend) of the pre- and post-spacer CT scans with the prostate CTV Tpsv (red), urethra , urethra_PRV (both yellow) the rectum (cyan) and the spacer in magenta for patient 7 and 12. The dotted lines indicate the structures on post-spacer CT scan. The location of the DIL is indicated with the red star**


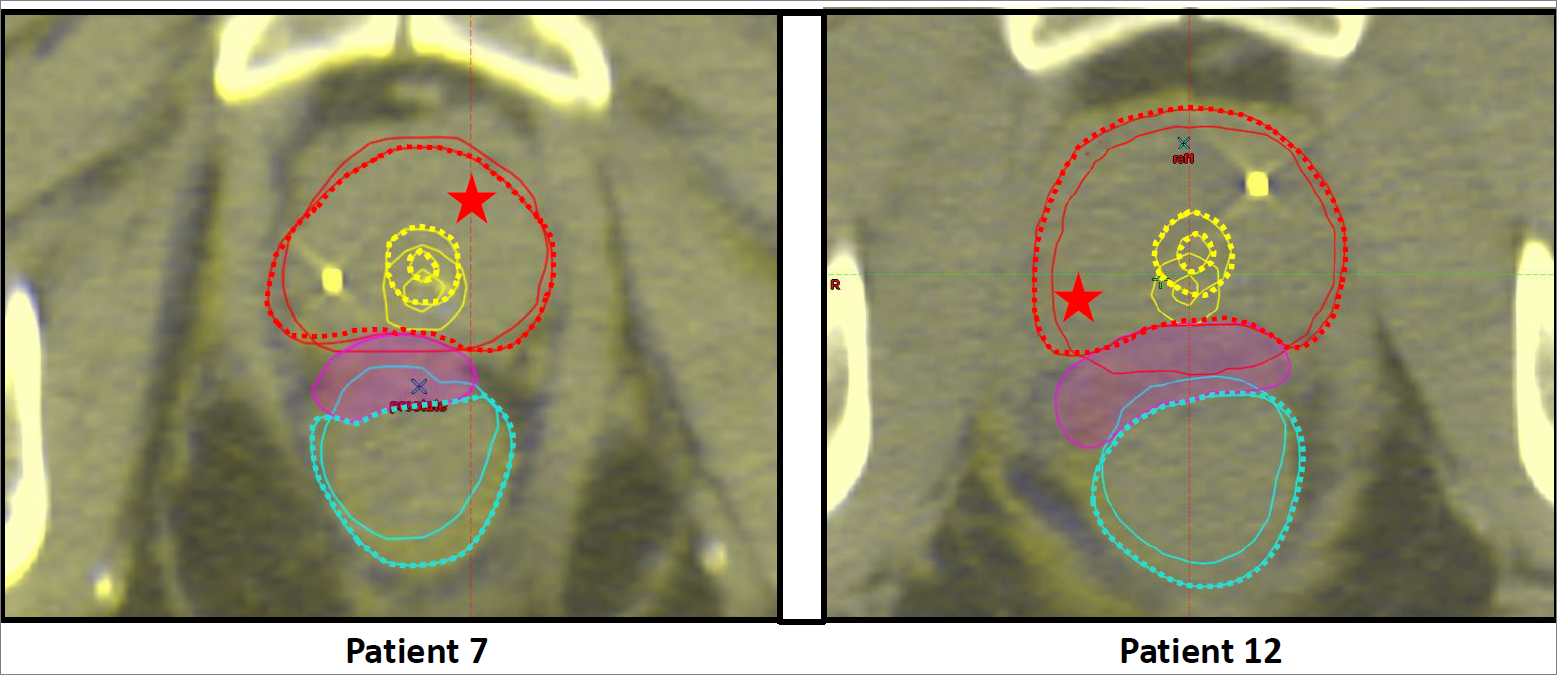


**Supplementary Figure SF4: Example of spacer position relative to the PTV, CTVpb and OARs in three patients. For patient two, the boost volume was in the apex of the prostate where the spacer gel was not present (spacer was inserted at the base and mid-level of the prostate but was missing in lower part of the apex).**


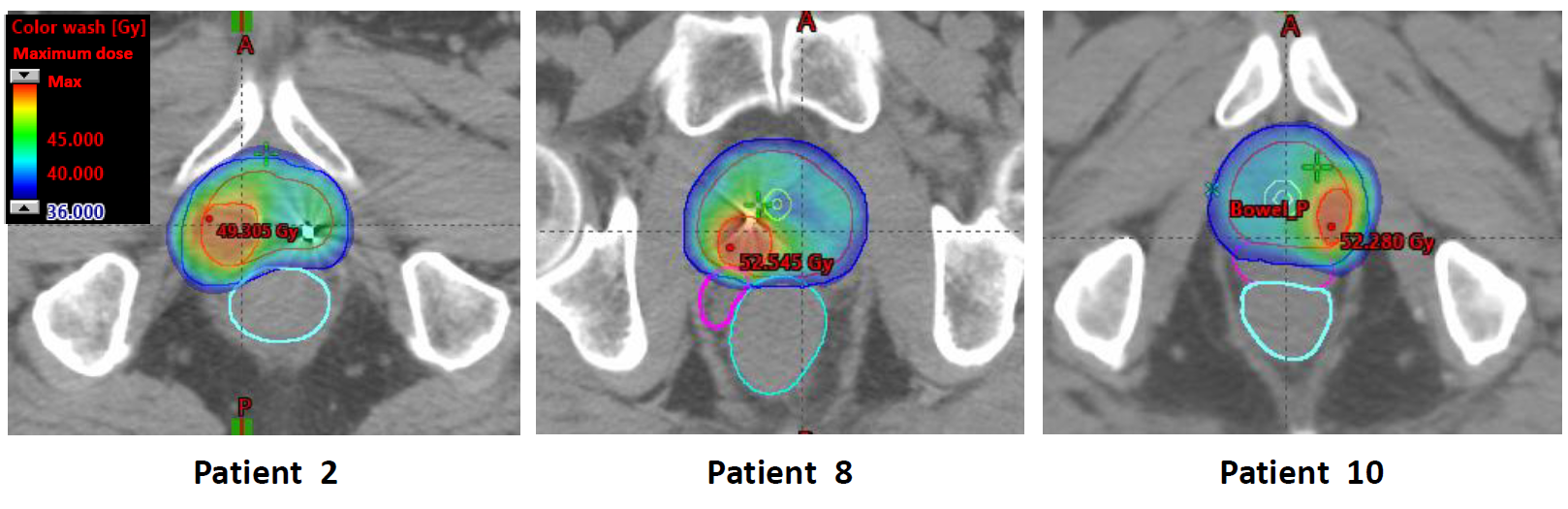

Supplement: Supplementary file 1 — Additional file 1. Supplementary material. [file 13014_2022_2003_MOESM1_ESM.docx]
